# Stress ulcer prophylaxis versus placebo—a blinded randomized control trial to evaluate the safety of two strategies in critically ill infants with congenital heart disease (SUPPRESS-CHD)

**DOI:** 10.1186/s13063-020-04513-w

**Published:** 2020-06-29

**Authors:** Kimberly I. Mills, Ben D. Albert, Lori J. Bechard, Christopher P. Duggan, Aditya Kaza, Seth Rakoff-Nahoum, Hera Vlamakis, Lynn A. Sleeper, Jane W. Newburger, Gregory P. Priebe, Nilesh M. Mehta

**Affiliations:** 1 Department of Cardiology, Boston Children’s Hospital, Boston, MA USA; 2 Harvard Medical School, Boston, MA USA; 3 Division of Critical Care Medicine, Department of Anesthesiology, Critical Care and Pain Medicine, Boston Children’s Hospital, Boston, MA USA; 4 Center for Nutrition, Boston Children’s Hospital, Boston, MA USA; 5 Department of Cardiac Surgery, Boston Children’s Hospital, Boston, MA USA; 6 Department of Pediatrics, Boston Children’s Hospital, Boston, MA USA; 7 Broad Institute of MIT and Harvard, Boston, MA USA

**Keywords:** Pediatric critical care, Pediatric cardiac critical care, Pediatric intensive care, H2 blocker, Congenital heart disease, Gastrointestinal hemorrhage, Infection, Microbiome

## Abstract

**Background:**

Critically ill infants with congenital heart disease (CHD) are often prescribed stress ulcer prophylaxis (SUP) to prevent upper gastrointestinal bleeding, despite the low incidence of stress ulcers and limited data on the safety and efficacy of SUP in infants. Recently, SUP has been associated with an increased incidence of hospital-acquired infections, community-acquired pneumonia, and necrotizing enterocolitis. The objective of this pilot study is to investigate the feasibility of performing a randomized controlled trial to assess the safety and efficacy of withholding SUP in infants with congenital heart disease admitted to the cardiac intensive care unit.

**Methods:**

A single center, prospective, double-blinded, randomized placebo-controlled pilot feasibility trial will be performed in infants with CHD admitted to the cardiac intensive care unit and anticipated to require respiratory support for > 24 h. Patients will be randomized to receive a histamine-2 receptor antagonist (H2RA) or placebo until they are discontinued from respiratory support. Randomization will be performed within 2 strata defined by admission type (medical or surgical) and age (neonate, age < 30 days, or infant, 1 month to 1 year). Allocation will be a 1:1 ratio using permuted blocks to ensure balanced allocations across the two treatment groups within each stratum. The primary outcomes include feasibility of screening, consent, timely allocation of study drug, and protocol adherence. The primary safety outcome is the rate of clinically significant upper gastrointestinal bleeding. The secondary outcomes are the difference in the relative and absolute abundance of the gut microbiota and functional microbial profiles between the two study groups. We plan to enroll 100 patients in this pilot study.

**Discussion:**

Routine use of SUP to prevent upper gastrointestinal bleeding in infants is controversial due to a low incidence of bleeding events and concern for adverse effects. The role of SUP in infants with CHD has not been examined, and there is equipoise on the risks and benefits of withholding this therapy. In addition, this therapy has been discontinued in other neonatal populations due to the concern for hospital-acquired infections and necrotizing enterocolitis. Furthermore, exploring changes to the microbiome after exposure to SUP may highlight the mechanisms by which SUP impacts potential microbial dysbiosis of the gut and its association with hospital-acquired infections. Assessment of the feasibility of a trial of withholding SUP in critically ill infants with CHD will facilitate planning of a larger multicenter trial of safety and efficacy of SUP in this vulnerable population.

**Trial registration:**

ClinicalTrials.gov, NCT03667703. Registered 12 September 2018, https://clinicaltrials.gov/ct2/show/NCT03667703?term=SUPPRESS+CHD&draw=2&rank=1.

All WHO Trial Registration Data Set Criteria are met in this manuscript.

## Background

Stress ulcer prophylaxis (SUP) is prescribed in the critically ill to decrease the incidence of stress-related mucosal damage that can lead to upper gastrointestinal (UGI) bleeding. The development of an UGI bleed while critically ill has been associated with increased intensive care unit (ICU) length of stay and mortality [1]. The adult practice of SUP administration has subsequently been adopted in neonatal and pediatric ICUs. However, the incidence of clinically significant UGI bleeds is very low, and there are limited data on the efficacy of SUP in critically ill infants and children [2]. Furthermore, the safety and efficacy of SUP has been called into question even in adults over the last decade [3–8].

In both adults and children, the incidence of clinically significant UGI bleeding is declining and hypothesized to be related to earlier initiation of enteral nutrition and increased utilization of goal-directed therapies [9]. Therefore, focus has shifted away from the utility of SUP and onto the safety and potential benefits of withholding this therapy. In critically ill adults, SUP has been associated with ventilator-associated pneumonia and *Clostridioides difficile* colitis [10, 11]. Similarly, in a large multicenter cohort, we reported a significant association between SUP and ventilator-associated pneumonia in critically ill children [8]. Several publications have also described an increase in bacteremia, necrotizing enterocolitis, and mortality in the neonatal ICU when patients were exposed to SUP [3, 5, 6]. These earlier studies have been limited by observational study design and potential confounding by indication. A definitive trial comparing administration versus withholding of SUP in critically ill infants with CHD is lacking. Based on the findings of prior studies, the role of SUP is being questioned [4, 12]. Furthermore, recent advances in molecular methods have allowed a shift from traditional culture-based techniques to detect gut microbiota to high-throughput DNA sequencing methods. The impact of acid suppression on the gut microbiota, especially the change in proportion of unfavorable organisms such as *C. difficile* and/or aerobic Gram-negative bacilli, may be an important mechanistic link between SUP and increased risk of infections.

Critically ill infants with congenital heart disease (CHD) are at risk for stress-related mucosal damage due to reduced splanchnic blood flow leading to mucosal ischemia and reperfusion injury, yet there is currently no consensus among pediatric cardiac intensivists regarding the clinical indications for SUP [13]. Therefore, the central objective of our study is to investigate the feasibility of conducting a clinical trial to assess the safety of withholding SUP in infants with CHD in the cardiac intensive care unit. Our hypothesis is that this trial design is feasible. We will also examine serial changes in the gut microbiota as a secondary aim to examine the difference in microbial profiles of patients receiving acid suppressive therapy compared to those receiving placebo. Our hypothesis is that patients receiving SUP will have decreased abundance and heterogeneity of gastrointestinal microbiota. Results of this feasibility trial will allow us to further refine inclusion and exclusion criteria, study procedures, data acquisition strategy, and study outcomes for a future multicenter, randomized controlled trial.

## Methods

### Study design

The study is a prospective, double-blinded randomized placebo-controlled pilot feasibility trial in a pediatric cardiac ICU at Boston Children’s Hospital, a quaternary freestanding children’s hospital. Enrollment began in February 2019 with a planned enrollment of 100 patients over a 2-year period. The local Institutional Review Board (IRB) at Boston Children’s Hospital approved this study. All study protocol amendments, deviations, or adverse events will be immediately reported to IRB. All research team members, clinicians, data analysts, and trial participants are blinded to study assignments. Unblinding will only occur in the event a participant has a serious adverse event such a clinically significant gastrointestinal bleed. The study was registered at ClinicalTrials.gov (NCT03667703) and funded by The Gerber Foundation’s National Research Grant (#5781). The funding agency will have no access to or involvement in the data analysis or writing of the manuscripts. The research integrity, data quality, and adverse event assessment will be regularly reviewed by a pre-appointed, independent Data Safety Monitoring Board (DSMB). The DSMB has an appointed chair and five other members. Further details regarding the charter can be available by contacting the corresponding author who is a co-principal investigator. Table 1 shows the Standard Protocol Items: Recommendation for Interventional Trials (SPIRIT) schedule for the enrollment, intervention, and assessment periods. The SPIRIT checklist is in Additional file 1.

**Table 1:** Standard Protocol Items: Recommendation for Interventional Trials (SPIRIT) schedule of enrollment, interventions, and assessments

Time point			Study period			
		Enrollment	Allocation		Post-allocation	Close-out
		Pre-study	Study day #1	Study day #2 through up to 14	24 h after respiratory support discontinued	Hospital discharge
Enrollment	Eligibility screen	**♦**	**♦**			
	Informed consent	**♦**	**♦**			
	Allocation		**♦**			
Intervention	H2 blocker or placebo		**♦**	**♦**		
Assessments	Demographics		**♦**	**♦**	**♦**	
	Laboratory data		**♦**	**♦**	**♦**	
	Antibiotic exposure		**♦**	**♦**	**♦**	
	Inotrope and vasoactive use		**♦**	**♦**	**♦**	
	Nutritional support		**♦**	**♦**	**♦**	
	Gastrointestinal bleeding		**♦**	**♦**	**♦**	**♦**
	Necrotizing enterocolitis		**♦**	**♦**	**♦**	**♦**
	Infectious complications		**♦**	**♦**	**♦**	**♦**
	Adverse event		**♦**	**♦**	**♦**	**♦**
	Oral, gastric, blood, and urine samples		**♦**		**♦**	**♦**
	Stool samples		**♦**	**♦**	**♦**	**♦**
	Length of stay					**♦**
	Mortality					**♦**

### Study population

The inclusion and exclusion criteria are described in Table 2. Infants diagnosed with CHD admitted to the cardiac ICU and anticipated to require respiratory support (defined below) for greater than 24 h will be eligible for the study. Congenital heart disease includes anatomic, myopathic, and arrhythmic conditions. Respiratory support is defined as mechanical ventilation, including conventional, high frequency oscillatory, or jet ventilation, as well as non-invasive positive pressure ventilation, such as continuous (CPAP) and biphasic (BIPAP) positive airway pressure, and high-flow nasal cannula. Respiratory support for greater than 24 h was chosen as a surrogate for severity of illness. Patients will be excluded if they receive any form of antacid for > 7 days during the past month as this could potentially alter their baseline gut microbiome. Patients will also be excluded if they are anticipated to receive high-dose steroids, intravenous non-steroidal anti-inflammatory agents, or high-dose aspirin during their hospitalization, as these medications may potentially cause gastritis and increase the risk for UGI bleeding. Finally, infants on certain anticoagulants—direct thrombin inhibitors and GPIIbIIIa inhibitors—will be excluded since these medications do not have an available reversal agent in the event of an UGI bleed.

**Table 2:** Eligibility criteria

**Inclusion criteria**	(1) < 12 months of age (including premature newborns) (2) Diagnosed with congenital heart disease* (3) Admitted to the CICU (4) Anticipated to require respiratory support^¶^ for > 24 h during their CICU stay (5) Have received ≤ 1 dose of stress ulcer prophylaxis^β^ during their current admission
**Exclusion criteria**	(1) Prior use of antacids^β^ in the past month for > 7 days (2) Active gastrointestinal bleeding (3) Active *Helicobacter pylori* infection (4) Anticipated exposure to certain pharmaceuticals: (i) High-dose steroids (equivalent to 4 mg/kg/day of methylprednisolone) (ii) Intravenous non-steroidal anti-inflammatory drugs (i.e., ketorolac) (iii) Certain anticoagulants including high-dose aspirin, direct thrombin inhibitors, and GPIIbIIIa inhibitors (5) Planned to undergo or recently has undergone gastrointestinal surgery within the last 4 weeks (6) Supported by ECMO or VAD (7) Currently enrolled in another conflicting interventional trial (8) Known to be allergic to H2RAs (9) Admitted for palliative care (10) Prior enrollment in the study (11) Primary provider declines enrollment

*CICU* cardiac intensive care unit, *H2RA* histamine-2 receptor antagonist, *PPI* proton pump inhibitor, *GIIbIIIa* glycoprotein IIb/IIIa, *ECMO* extracorporeal membrane oxygenator, *VAD* ventricular assist device

*CHD includes anatomic, myopathic, and arrhythmic conditions

^¶^Respiratory support includes mechanical ventilation, non-invasive positive pressure ventilation, and high-flow oxygen therapy

^β^Stress ulcer prophylaxis or antacids include H2RAs, PPIs, and sucralfate

### Study setting

The study will be conducted in the cardiac intensive care unit at Boston Children’s Hospital, a quaternary referral center and standalone children’s hospital.

### Recruitment and study flow

**Fig. 1:**
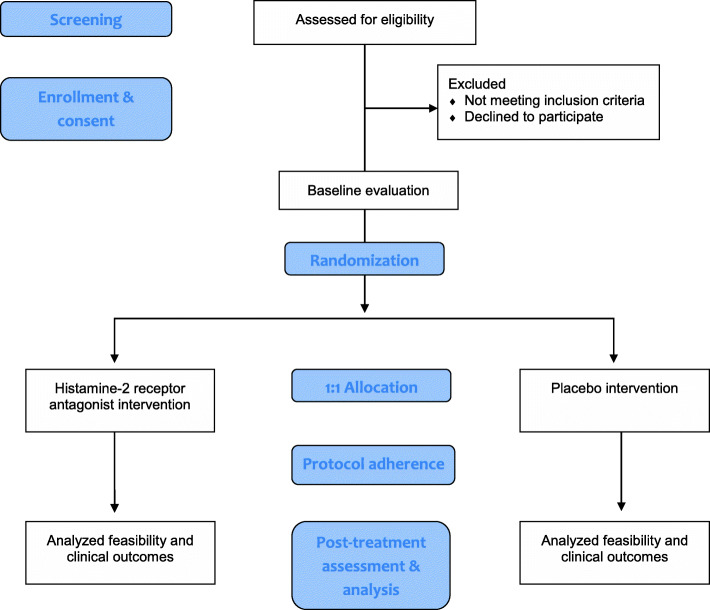
Study flowchart of trial design

Potential patients will be screened for eligibility, and one of the principal investigators will approach the family of an eligible patient for written consent. If the legal guardian grants consent, the patient will be enrolled in the study and then randomized to one of two arms. Please see consent form in Supplementary files. Study procedures will be continued until the patient (1) no longer requires respiratory support for greater than 24 h, (2) transfers to the floor or is discharged from the cardiac ICU, (3) completes 14 days of study drug, or (4) at any time during the study the primary provider believes that open-label acid suppression is indicated (Fig. 1). To ensure adequate enrollment and retention, we will provide education of the study objectives and procedures to important subspecialist groups, send mailers to eligible patients prior to delivery or surgery, and post signs about the study in the cardiac intensive care unit and preoperative clinic.

### Randomization

Eligible patients will be randomly assigned by Boston Children’s Research Pharmacy to receive either a histamine-2 receptor antagonist (i.e., ranitidine or famotidine per institutional standard) or placebo. The randomization assignments are generated by Boston Children’s Hospital’s proprietary randomization software, SciRan®. Randomization will be performed within 2 strata defined by admission type (medical or surgical) and age (neonate, age < 30 days, or infant, 1 month to 1 year). Allocation will be using permuted blocks in a 1:1 ratio to ensure balanced allocations across the two treatment groups within each stratum. Allocation concealment is achieved by ensuring that only the pharmacy team holds the randomization key. The pharmacy does not partake in the outcome variable assessment, and the study investigators do not have access to the randomization key.

### Sample size

We powered the precision of our feasibility estimates for both screening and drug initiation—which we deemed as the two most important feasibility measures. There are approximately 600 patients under 1 year of age admitted to the cardiac ICU each year, and we anticipate that an estimated 200 patients will be eligible over the 2-year recruitment period. The target to demonstrate feasibility is screening 80% of all patients. If *n* = 600, the lower limit of the 95% one-sided confidence interval will include 80% as long as the observed screening rate is at least 82.7%. There are 100 patients to be randomized in this pilot trial. The target to demonstrate feasibility with respect to drug initiation is to have 80% of randomized patients receive their first dose of study drug within 48 h. With *n* = 100, the lower limit of the 95% one-sided confidence interval will include 80% as long as the observed drug initiation rate is at least 86.9%. That is, as long as the observed drug initiation rate is at least 86.9%, we can be 95% confident that the drug initiation rate, to be realized in a future trial, is at least 80%. The study is not powered to assess a statistical difference between the incidence of UGI bleeding and hospital-acquired infections, as the historical incidences are very low, 0.5% and 2%, respectively. These outcomes will be further assessed in a future larger, multicenter trial.

For the assessment of oral, gastric, and stool microbiota, a total of 600 samples (2 samples per site) will be obtained from the 100 patients. We estimate detectable effect sizes using these sample sizes based on the Human Microbiome Project [14]. With adjustment for three covariates: one categorical binary (antibiotics), one continuous (age), and one categorical tertiary (hospital-acquired infections), we anticipate a power of 0.9 to detect a 1.36% level of rare taxon relative abundance and a 3.46% level of common taxa relative abundance, within a treatment arm between two time points, and similarly between two treatment arms at a given time point for either oral, gastric, or stool samples. All estimates incorporate stringent Bonferroni multiple-hypothesis correction to an adjusted *p* value of 0.05. Approximating as above, the corresponding detection limit for 250 metagenomic pathways with power of 0.9 would be 3.87%.

### Study intervention

Study participants will be randomly assigned to receive a histamine-2 receptor antagonist (i.e., ranitidine or famotidine) or placebo. As there are no pediatric-specific recommendations regarding type of SUP, we elected to conduct our study with a histamine-2 receptor antagonist since this is the current standard practice at our institution. In this pragmatic design, participants can receive study drug either intravenously or enterally, depending on the clinician’s preference. The dosing is based on age and route. For ranitidine, neonates (< 31 days old) will receive either 1 mg/kg intravenously/enterally every 12 h, and infants (≥ 31 days old) will receive 1 mg/kg intravenously/enterally every 8 h. For famotidine, if the participant is < 3 months, they will receive 0.5 mg/kg/dose IV/PO daily, and if > 3 months old, they will receive 0.25 mg/kg/dose IV every 12 h or 0.5 mg/kg/dose PO every 12 h. As part of pharmacovigilance, surveillance for hepatic dysfunction and thrombocytopenia will be included in the case report forms.

The placebo will be in an equivalent volume of 0.9% saline intravenously or a stevioside sugar-free syrup vehicle that resembles the color, tonicity, and texture of oral ranitidine or famotidine. Individualized unit-dose syringes will be provided to each study participant and blinded to the study team, bedside clinicians, parents, and outcome assessors. All interventions other than the study drug are left to the discretion of the treating clinicians. Once a participant completes the study, the clinicians can prescribe acid suppression per their usual practice. Adherence to intervention protocols is monitored daily by research staff by checking in with participant’s bedside nurse to ensure medication administration and timely sample collection. The research staff also speaks with the attending physician daily to assess for any adverse events or study-related issues. Patients with hemodynamically significant UGI bleeding will be withdrawn from the study, as they would likely require open-label acid suppression. There also exist study-halting criteria, which would stop the study until the DSMB reviewed and recommended continuation of the study. Final determination of trial termination will be made by principal investigators, if necessary. Auditing of trial conduct is done every 6 months by the DSMB, independent of the investigators and sponsor. The study-halting criteria include a total of 3 UGI bleeding events or an enrollment number of less than 20 patients per year. The principal investigators will have access to the final trial dataset.

### Measurements and definitions

Data will be imported into a secure, password-protected, FDA-compliant database (InForm® Electronic Data Capture). Data collection will include demographic, procedural, laboratory, pharmaceutical, nutritional, ventilatory, and outcome variables. Samples obtained will include oral swabs, gastric aspirates (via indwelling NG tube), discarded blood, and urine at the initiation and conclusion of the study. In addition, serial stool samples will be collected while on study. Data are collected in daily case report forms (CRFs) and then imported weekly into the InForm ITM (Integrated Trial Management) System. Monthly audits of the InForm database are completed with the study investigators to ensure completeness and minimize transcription errors. In addition, the InForm database has safety metrics built in for out of bounds values.

Important definitions include the following:

1. Clinically significant UGI bleed—new-onset bleeding from the UGI tract (i.e., hematemesis, bloody gastric aspirate, or hematochezia) AND associated with (a) decrease in hemoglobin by 2 g/dL, OR (b) decline in mean arterial blood pressure by 10 mmHg or initiation/increase of inotrope/vasoactive medications, OR (c) increase in heart rate by 20 beats per minute in the absence of an arrhythmia or fever, OR (d) need for unanticipated blood transfusion, OR (e) unexpected endoscopic or operative procedure to achieve hemostasis. This definition has been used in adult randomized controlled trials with excellent inter-rater agreement [15, 16].
2. Bloodstream infection (BSI)—a laboratory-confirmed bloodstream infection with or without a central line in place.
3. Ventilator-associated event (VAE)—a deterioration in respiratory status after a period of stability or improvement on the ventilator, evidence of infection or inflammation and laboratory evidence of a respiratory infection (CDC).
4. Urinary tract infection—a laboratory-confirmed urinary tract infection with or without a urinary catheter in place.
5. *Clostridium difficile* associated diarrhea—diarrhea in the presence of a positive *C. difficile* test.
6. Mediastinitis—a laboratory-confirmed organism from mediastinal tissue or fluid, or based on gross anatomic exam, or has hyper/hypothermia or apnea or bradycardia or sternal instability with at least one of the following: (a) purulent drainage from the mediastinal area or (b) mediastinal widening on imaging (CDC).
7. Superficial wound infection—has two of the following symptoms: (a) erythema, (b) tenderness, (c) swelling AND a laboratory-confirmed organism is identified from the wound.
8. Gastrointestinal microbiota—difference in oral and stool microbiome between the 2 groups in this study will be examined.

### Sample analyses

For assessment of stool microbiota, sequence-based microbial community surveys of stool samples will be carried out by 16S rRNA gene-based sequencing in the Microbial Genomics and Transcriptomics Core at the Broad Institute. Their protocol targets the V4 window of the 16S rRNA gene and uses Illumina MiSeq system at the Broad Genomics Platform to produce on average 25,000 quality filtered, stitched paired-end reads per sample, representing the current state-of-the-art [17]. In addition, a higher resolution community survey and complementary functional survey of a subset of the stool samples (20%) will be obtained using metagenomic shotgun DNA sequencing, to be performed at the Broad Institute in the Broad Technology Labs and Genomics Platform. Metagenomic libraries will be constructed using the Nextera XT DNA library preparation kit (Illumina) and sequencing will be performed on an Illumina HiSeq 2000 to generate a minimum of 2 Gb of 101 nt paired-end reads.

For evaluation of oral and gastric microbiota, samples will be subjected to ribosomal DNA (rDNA) amplicon sequencing to characterize the composition of these microbiome communities. First, DNA will be extracted using a robust commercial extraction kit. Next, rDNA hypervariable regions of specific kingdoms will be amplified using universal primers. For bacteria, 16S V3V4 will be targeted using 5′-CCTACGGGNGGCWGCAG-3′ and 5′-GGACTACNVGGGTWTCTAAT-3′; for fungi, ITS1 is targeted using 5′-CTYGGTCATTTAGAGGAAGTAA-3′ and 5′-GCTGCGTTCTTCATCGATGC-3′. Specifically, Illumina adapter sequences and variable spacers are added 5′ upstream of these sequences to enable high-throughput multiplexed sequencing. The prepared amplicons will then be pooled and sequenced using Illumina Miseq 2 × 300 bp platform. Illumina raw reads will be de-multiplexed, quality trimmed, dereplicated and denoised, and finally mapped against established rDNA databases. The derived OTU table will document the relative abundance of each taxonomy within each sample.

### Outcomes

The central objective for our pilot study is to investigate whether a clinical trial assessing the safety and efficacy of withholding SUP in infants with CHD in the cardiac ICU is feasible. The trial will be considered feasible if each of the following 4 a priori variables are met: (1) > 80% of eligible patients are approached for consent (*screening*), (2) > 20% of eligible patients are randomized (*enrollment and consent*), (3) > 80% of consented patients received their first dose of study drug within 48 h (*allocation*), and (4) > 80% protocol compliance achieved (*protocol adherence*). Adherence to the protocol is defined as having received all doses of study drug as prescribed during the study period. Protocol deviation is defined as either premature termination of the study or prescription of SUP that is not part of the study while enrolled. In addition to feasibility, we will assess safety by comparing the difference in the incidence of clinically significant UGI bleeding and hospital-acquired infections between participants receiving SUP versus placebo. Finally, we will explore the changes to the gut microbiota by comparing the absolute and serial differences in the abundance of bacteria and functional microbial profiles between those receiving SUP compared to placebo. The study investigators will not be blinded to primary outcome measures. The microbiome specimens (for secondary outcome measure) will be processed and analyzed without revealing their study group allocation and thus will be blinded.

### Statistics

The study will be double-blinded, and we will utilize an intention-to-treat (primary analysis) and per protocol (secondary) analysis. Trial participants who do not complete the intervention will remain in the primary, intention-to-treat analysis of trial outcomes. Data will be reviewed every 6 months by the DSMB. Homogeneity of the two treatment arms will be assessed using a Fisher exact or chi-square test for categorical variables and a Student *t* test (parametric) or Wilcoxon rank sum test (non-parametric) for continuous variables.

#### Feasibility analysis

For each feasibility outcome measure, we will report the proportions of screened patients and/or participants meeting each criterion successfully and the associated one-sided 95% confidence interval.

#### Safety analysis

A two-sided 95% confidence interval will be constructed for treatment difference in the proportion of patients with UGI bleeding and hospital-acquired infections, as well as for each treatment-arm specific rate of UGI bleeding.

#### Microbiome analysis

Once the rRNA sequencing is completed, we will perform taxonomic profiling to identify distinct microbial lineages and then compare them to the published Greengenes, SILVA (for 16S), and UNITE (for ITS1) Reference Database [18–20]. The primary characteristics to be assessed between treatment arms are as follows: (a) within-sample and between-sample overall ecology of the microbial community, (b) absolute and relative abundance of microbial communities, and (c) pattern classification analysis to identify diversity [21]. We will then perform per-feature multivariable association analyses that estimate which microbiome attributes differ by treatment arm as well as between time points, while accounting for covariates, to identify how microbes are affected by outcomes in the presence of certain covariates [22–24]. We will adjust for delivery type (C-section vs. vaginal delivery), nutrition type (breast milk vs. formula), and antibiotics prescribed to the patient (not mother) as these are known confounders in the infant gut microbiome.

## Discussion

The practice of routine acid suppressive therapy in infants admitted to the CICU must be examined. Rising concerns related to the adverse effects of acid suppression, particularly hospital-acquired infections, must be acknowledged and the indications for this therapy need to be revisited. Therefore, we have proposed a pilot feasibility trial to explore the safety of withholding SUP and examine the changes to the microbiome after exposure to SUP in this population. Our trial will highlight the mechanisms by which SUP impacts the microbial dysbiosis of the gut and its association with hospital-acquired infections. If withholding of SUP is deemed safe and associated with less disruption of the gut microbial profile, it could guide a significant change in practice in centers where this therapy is routinely prescribed.

Although considered the gold standard, there are several barriers to conducting high-quality randomized controlled trials in pediatric critical care. Included in these barriers are scarcity of research funding and failure to complete the trial due to difficulty with patient enrollment in the ICU environment [12]. Given these hurdles, large randomized controlled trials are sparse in pediatric critical care and several trials have been stopped early, most commonly due to futility [25, 26]. To address the challenges with timely completion of a trial, certain governing bodies explicitly recommend that feasibility and pilot studies be conducted prior to undertaking and funding larger randomized controlled trials [27]. At the conclusion of our feasibility trial, we will be able to identify important barriers and, if necessary, amend the study protocol in order to proceed with a larger randomized controlled trial in the future. If this pilot trial concludes that a larger trial is feasible, we plan to conduct a non-inferiority, multicenter randomized controlled trial in pediatric cardiac ICUs focusing on clinical outcomes shortly thereafter. We hypothesize that withholding SUP in critically ill infants with CHD does not increase the incidence of UGI bleeding and results in less disruption of the gut microbiota, possibly decreasing the incidence of hospital-acquired infections, compared to SUP therapy. The results of our study will potentially define appropriate indications for SUP in infants admitted to the CICU and illuminate the mechanistic relationship between microbial alterations and outcomes during critical illness.

There are a few limitations we have identified in our current study protocol. First, the anticipated duration of respiratory support of more than 24 h was chosen as an enrollment criterion because it will exclude patients with short ICU stays and exposure to the intervention. To examine whether anticipated duration of respiratory support is a reasonable marker for length of stay and exposure to the intervention, we plan to compare actual duration of respiratory support and length of stay in those who screened in versus those who screened out. Second, obtaining gastric samples may be difficult in participants as infants often make very little gastric fluid when critically ill. Finally, some infants might not have regular bowel movements, limiting the frequency of stool samples and thereby limiting the longitudinal or treatment effect assessment.

## Trial status

The submitted protocol is the 3rd version, last amended on October 17, 2019. Recruitment for the study began on February 4, 2019, and should be completed by January 2021. The recent recall of ranitidine prompted removal of that drug from the institutional formulary, and famotidine will be the histamine-2 antagonist for this study.

## Supplementary information

**Additional file 1.** SPIRIT 2013 Checklist: Recommended items to address in a clinical trial protocol and related documents.

## Data Availability

The results of the trial are the whole property of the investigators and the institution and will be published in scientific journals without restriction. The datasets used and/or analyzed during the current study are available from the corresponding author on reasonable request.

## Abbreviations

H2RA: Histamine-2 receptor antagonist
UGI: Upper gastrointestinal
SUP: Stress ulcer prophylaxis
ICU: Intensive care unit
CHD: Congenital heart disease
IRB: Institutional Review Board
DSMB: Data Safety Monitoring Board
SPIRIT: Standard Protocol Items: Recommendation for Interventional Trials
CPAP: Continuous positive airway pressure
BIPAP: Biphasic positive airway pressure
